# Extinction of Contextual Fear Memory and Passive Avoidance Memory and Subsequent Anxiety-like and Depressive-like Behavior of A53T and A53T-L444P Mice

**DOI:** 10.3390/genes16091004

**Published:** 2025-08-26

**Authors:** Emily Bunnell, Elizabeth Saltonstall, Alexandra Pederson, Charlie Baxter, Elia Ramicciotti, Naomi Robinson, Phoebe Sandholm, Abigail O′Niel, Jacob Raber

**Affiliations:** 1 Department of Behavioral Neuroscience, L470, Oregon Health & Science University, 3181 SW Sam Jackson Park Road, Portland, OR 97239, USA; bunnelle@ohsu.edu (E.B.); saltonse@ohsu.edu (E.S.); pedersoa@ohsu.edu (A.P.); baxterc@ohsu.edu (C.B.); eramacciotti@linfield.edu (E.R.); robinnao@ohsu.edu (N.R.); phoebesand816@gmail.com (P.S.); oniela@ohsu.edu (A.O.); 2 Departments of Neurology, Psychiatry, and Radiation Medicine, Division of Neuroscience ONPRC, Oregon Health & Science University, Portland, OR 97239, USA; 3 College of Pharmacy, Oregon State University, Corvallis, OR 97331, USA

**Keywords:** GBA, A53T, A53T-L444P, Parkinson’s, PTSD, fear conditioning, avoidance

## Abstract

**Background:** Genetic factors pertinent to Parkinson’s disease (PD) might predispose an individual to post-traumatic stress disorder (PTSD). Humans who are heterozygous for the glucocerebrosidase 1 (GBA) L444P Gaucher mutation have an increased PD risk and elevated levels of alpha synuclein (aSyn). Mice that are heterozygous for the GBA mutation and express aSyn with the A53T mutation show elevated anxiety levels at 20 months of age compared to those expressing only A53T. **Objective:** This study aims to assess whether A53T and A53T-L444P affect the risk of developing PTSD phenotypes and whether sex and age modulate this risk. **Methods:** Young (5.1 ± 0.2 months) and older (11.3 ± 0.2 months) A53T and GBA L444P female and male mice were tested for fear learning and memory extinction in the contextual fear conditioning and passive avoidance paradigms. Subsequently, the mice were tested for measures of activity and anxiety in the open field and for depressive-like behavior in the forced swim test. **Results:** In the contextual fear memory extinction paradigm, only young A53T female mice showed contextual fear memory extinction, while older A53T female mice showed increased activity levels over subsequent days. In the passive avoidance memory paradigm, no mice showed extinction of passive avoidance memory. When the frequency of entering the more anxiety-provoking center of the open field was analyzed, a test history x sex x age interaction was observed. In the forced swim test, test history affected the depressive-like behavior in mice trained; there was more depressive-like behavior in mice trained in the contextual fear memory extinction paradigm than in mice trained in the passive avoidance memory extinction paradigm. Moreover, there was an effect of age with more depressive-like behavior in older than in younger mice, and an effect of genotype with more depressive-like behavior in A53T-L444P compared to A53T mice. When cortical phosphorylated tau (pS 199) levels were analyzed, there was an effects of genotype, a sex x age interaction, and ant age x test history interaction. **Conclusions:** A53T and A53T-L444P affect the risk of developing PTSD phenotypes. Fear extinction test history, genotype, and age affect depressive-like behavior and tau pathology.

## 1. Introduction

Parkinson’s disease (PD) is a progressive neurodegenerative motor function disorder. There is increased recognition of non-motor features; cognitive impairments are especially prevalent, from mild deficits (mild cognitive impairments, PD-MCI) to severe dementia in over 80% of patients who have had the disease for more than 20 years [1]. Mild cognitive deficits are common in the early phase of PD. They impact quality of life, exacerbate caregiver stress, and can progress to dementia [2]. As PD progresses, motor symptoms worsen, and cognitive problems become more apparent.

Following exposure to trauma, some individuals develop post-traumatic stress disorder (PTSD), which includes intrusive recollection of trauma and avoidance of trauma-related cues [3,4]. Following re-exposure to environmental cues that were present during the traumatic event, affected individuals show impaired extinction of fear and often avoidance of trauma-related cues [5,6]. Avoidance behavior is protective; however, it can contribute to severe symptoms and might interfere with exposure-based therapy [6,7]. Protective avoidance can also be considered as an active coping mechanism [8,9] and serve an adaptive function that might even be beneficial if included as part of exposure therapy [10,11,12,13]. Living with PTSD may increase the risk of developing PD. A 2022 cohort study of 8336 people in Israel found those living with PTSD had a 1.48-fold increased risk of PD compared to people not living with PTSD [14]. The PD risk was higher in men older than 72 years of age. In addition, a cohort study of 7280 people in Taiwan revealed that those with PTSD had a 3.46-fold increased PD risk [15]. Further, a Veteran study in the US involving 176,871 PD patients revealed that PTSD patients had a 2.71-fold increased PD risk [16]. In the Israeli study, the association between PTSD and PD in elderly male patients remained significant following adjustments for depression and traumatic lifetime events [14]. Although, in that study, the increased PD risk was only seen in male patients, in the Taiwanese study, this risk was also seen in females [15]. Notably, the risk of developing PTSD is 2–3-fold higher in women than in men [17], while the risk of developing PD is 2-fold higher in men than women [18]. However, women with PD show higher rates of disease progression and mortality than men with PD [18], and PTSD might contribute to this sex difference.

Genetic factors pertinent to PD might increase the risk of developing PTSD and/or more severe PTSD. For example, *PARK2* is associated with PTSD in men [19]. Humans who are heterozygous for the glucocerebrosidase 1 (GBA) L444P Gaucher mutation have an increased PD risk [20,21]. GBA mutations, which are associated with an earlier age at onset, faster decline, and higher risk of developing psychosis or dementia, result in elevated aSyn levels [22]. Aggregated aSyn may further worsen the condition. Heterozygous mice with the L444P mutation knocked-into the mouse gene (GBA*^+/L444P^*; B6;129S4-*Gba^tm1RlpMmnc^*) have reduced GBA protein and enzymatic activity levels, increased aSyn levels in the midbrain, and are more susceptible than wild-type mice to motor deficits, neuroinflammation, and dopamine (DA) neuronal pathology following exposure to the neurotoxin (1-methyl-4-phenyl-1,2,3,6-tetrahydropyridine) MPTP [23]. Compared to mice expressing only A53T, mice with the combined aSyn A53T and GBA L444P genotype show a motor phenotype at 14 to 15 months of age and an anxiety phenotype at 20 months of age [24]. We rederived the A53T-L444P and A53T mouse lines generated by Dr. Nussbaum [24] and used these mice in the current study. Environmental challenges of these mouse lines differentially affected behavioral and cognitive performance [25]. It is unclear whether A53T or A53T-L444P are associated with PTSD phenotypes. In this study, we assessed whether A53T and A53T-L444P affect the risk of developing PTSD phenotypes in mutant mice.

The extinction of contextual or cued fear memory by exposing the humans or animals to the same environment or environmental cues that were present during the initial trauma, but without including the aversive stimulus or stimuli, is often used to study PTSD [26,27,28,29,30,31,32]. The inability to suppress conditioned fear responses is the hallmark of PTSD [33,34], and a lot has been learned about the extinction of conditioned fear using preclinical models of fear conditioning. One limitation of the contextual and cued fear conditioning paradigm is that the animal cannot avoid the context or cues associated with the initial trauma. This is a significant limitation as those affected by trauma who have developed PTSD often show avoidance of trauma-related cues [5,6] as part of active coping [8,9,10,11,12,13]. In the passive and active avoidance paradigms, animals can actively or passively escape the trauma associated with aversive cues. Although, generally, memory retention in the passive avoidance test is only assessed at one time point, Chen et al. assessed passive avoidance memory retention over several days [35], allowing for the assessment of the extinction of passive avoidance memory.

Cortical levels of tau, a microtubule protein involved in stabilizing microtubule and synaptic physiology [36], are associated with cognition in humans in Positron Emission Tomography (PET) studies [37]. Phosphorylation of tau is especially associated with cognitive injury [38]. In this study, we assessed whether A53T and A53T-L444P mice show genotype-, sex-, and age-dependent-impaired contextual fear memory and passive avoidance memory extinction. In addition, we assessed whether the history of having received fear conditioning and passive avoidance memory extinction differentially affects exploratory behavior, measures of anxiety, depressive-like behavior, and phosphorylated tau levels in the cortex.

## 2. Materials and Methods

### 2.1. Animals

A53T-L444P and A53T mice, generated by Dr. Nussbaum [24] and regenerated through cryo-recovery and genotyped as reported in [25], were used in this study. The A53T-L444P and A53T mice were all littermates. Wild-type littermates were not used due to the complexity of the genetic model, because the mice are not on a homozygous background strain, and the percent of wild-type mice out of all mice generated does not justify this including wild-type littermates.

The mice were maintained on a PicoLab Rodent Diet 20, no. 5053 (PMI Nutrition International, St. Louis, MO, USA) with food provided ad libitum. The female and male mice were tested for extinction of contextual fear memory or passive avoidance retention memory, followed by assessment of activity and anxiety levels in the open field and depressive-like behavior in the forced swim test. There were 214 mice in total: 119 male mice and 95 female mice; 135 were A53T mice, and 79 were A53T-L444P mice; 96 were young mice (5.1 ± 0.2 months), and 118 were older mice (11.3 ± 0.2 months). The mice were randomly assigned to either the contextual fear memory extinction or passive avoidance memory extinction paradigm. For the extinction of contextual fear memory, we used 57 male and 50 female mice, 66 A53T and 41 A53T-L444P mice, and 39 younger mice and 68 older mice. For the extinction of passive avoidance memory, we used 62 male mice and 45 female mice, 69 A53T mice and 38 A53T-L444P mice, and 57 younger mice and 50 older mice. Because these mice are not from a homozygous background, we also tested 30 C57BL/6J mice (15 female (3.90 ± 0.12 month) and 15 male (3.90 ± 0.12 month) mice) for the extinction of passive avoidance memory. The sample size was determined based on our earlier fear memory extinction data. The mice were behaviorally tested in the same room as they were housed. All procedures were approved by the Institutional Animal Care and Use Committees at OHSU and were in compliance with all Federal regulations. All experiments were performed by researchers blinded to the genotype and age of the mice. The code was only broken when all the data were analyzed.

### 2.2. Extinction of Contextual Fear

**Figure 1:**
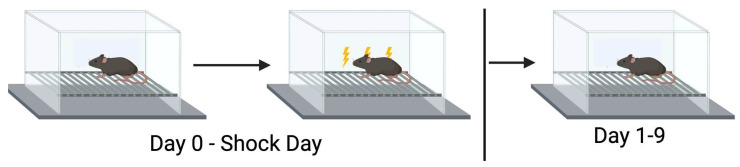
Extinction of contextual fear memory.

The contextual fear conditioning involved equipment from Med Associates ENV-022V Standard Expanded PVC, St. Albans, VT, USA) and Med Associates VideoFreeze software 2.5.4.0. (St. Albans, VT, USA) (Figure 1). The training trial lasted 5 min. There was a baseline period of 118 s received followed by a 2 s shock (0.5 mA). After a 118 s inter-stimulus interval, the mice received a second 2 s shock (0.5 mA) and remained in the enclosure for 60 s. The mice were placed back in the chamber for nine subsequent days for 300 s, and the percentage of time spent freezing and activity levels were analyzed. 

### 2.3. Extinction of Passive Avoidance Memory

**Figure 2:**
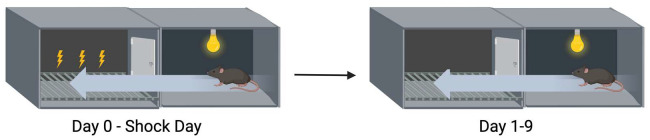
Extinction of passive avoidance memory.

The passive avoidance test involved a two-compartment chamber from Kinder Scientific (Poway, CA, USA) (Figure 2). Both compartments were dark at first. The mice were placed in one compartment of the chamber; after a 5 s period, a cue light turned on and the connecting gate opened. The mice received a mild foot shock (0.5 mA, 2 s) when they entered the dark compartment. On nine subsequent days, the mice were put back into the light compartment and no shock was delivered if they moved to the dark compartment. The trial ended if the mice entered the dark compartment within 300 s or up to 300 s if the mice did not enter the dark compartment. In the supplementary study with C57BL6/J mice, we did not include extinction days 5–8. The time to cross into the dark chamber and whether the mouse crossed over was recorded.

### 2.4. Open Field Test

**Figure 3:**
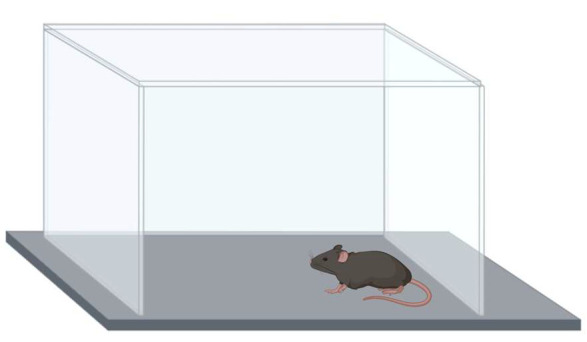
Activity and anxiety measures in the open field test.

On day 10, the mice were tested in an open field (Kinder Scientific, Poway, CA, USA) for 10 min (Figure 3). Activity levels (distance moved), time spent, and entries into the more anxiety-provoking center (8 × 8 inches) of the open field were analyzed using Noldus Ethovision video tracking software (version 17, Wageningen, The Netherlands).

### 2.5. Forced Swim Test

**Figure 4:**
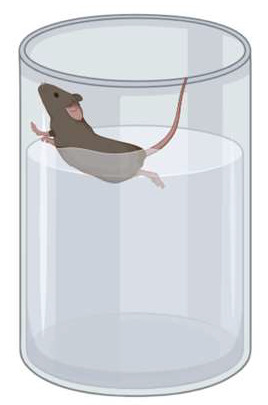
Depressive-like behavior in the forced swim test.

On day 11, the mice were tested for depressive-like behavior by placing them in a cylinder filled with water (water height: 15 cm; container diameter: 16–20 cm; 25 °C) for 6 min (Figure 4). Immobility—a cessation of limb movements except minor involuntary movements of the hind limbs or those movements necessary to stay afloat—was scored manually every 5 s, and the immobility percentage was calculated [39].

### 2.6. Phospoyrylated Tau Levels

Cortical levels of phosporylated tau (pS 199) were determined using the Invitrogen mouse tau pS 199 ELISA (KMB7041) (Waltham, MA, USA). The standard curve was run in duplicate and the samples as singles. The protein concentration of the samples was determined using the Bradford assay (BioRad, Hercules, CA, USA). Samples were diluted with PBS for the ELISA and stored at −80 °C until use. The samples were analyzed as follows: A53T mice: young fear conditioning: 5 females and 8 males; young passive avoidance: 8 females and 8 males; older fear conditioning: 11 females and 8 males; older passive avoidance: 5 females and 10 males. A53T-L444P mice: young fear conditioning: 3 females and 6 males; young passive avoidance: 6 females and 5 males; older fear conditioning: 10 females and 9 males; older passive avoidance: 8 females and 9 males.

### 2.7. Statistical Analyses

All behavioral data and the cortical phosphorylated tau levels were reported as mean ± standard error of the mean and were analyzed using SPSS v.22 (IBM, Armonk, NY, USA) or GraphPad v.10 (La Jolla, CA, USA) software. For the extinction of contextual fear memory and the extinction of passive avoidance memory, genotype, age, and sex, were included as factors in the analysis of variance (ANOVAs). For the open field and forced swim tests, history of test (i.e., fear conditioning or passive avoidance extinction paradigm) was included as a factor as well. If there were statistical interactions, ages, genotypes, or sexes were analyzed separately, as indicated. Repeated measures were used when appropriate. We also analyzed extinction for each extinction curve using ANOVA along with a Dunnett’s post hoc test comparing the freezing or latency percentages on days 2–9 to those on the first day of extinction. Statistical significance was considered as *p* < 0.05. When sphericity was violated (Mauchly’s test), Greenhouse–Geisser corrections were used. To assess relationships between phosphorylated tau levels and behavioral measures in the open field and forced swim test, Spearman correlational analyses were performed. All researchers were blinded to genotype and age, and the code was only broken after all the data were analyzed.

## 3. Results

### 3.1. Contextual Fear Learning

**Figure 5:**
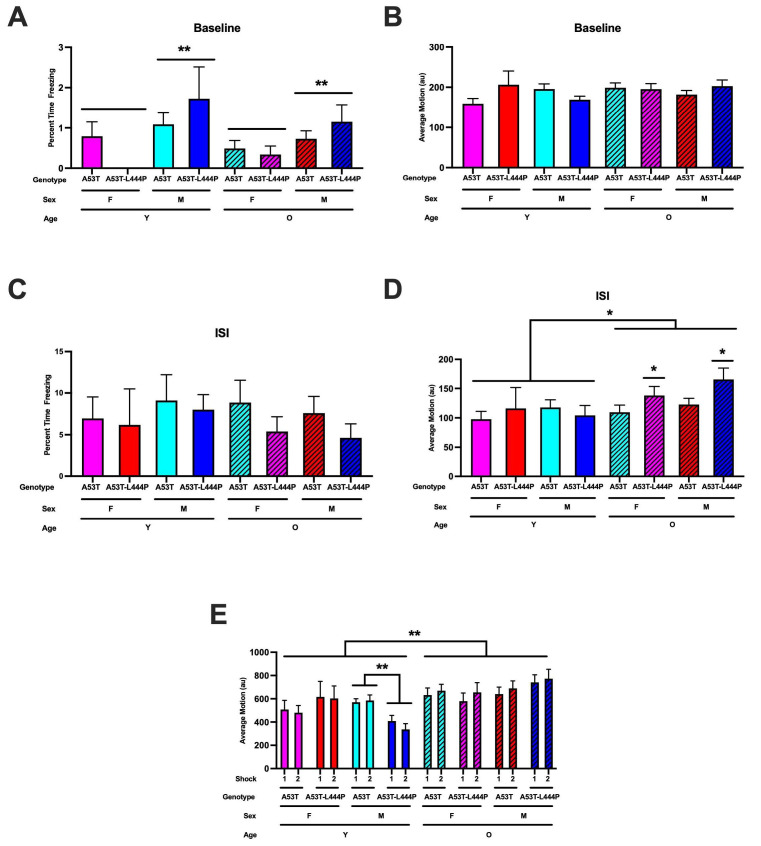
Percent freezing during the baseline period. (**A**). There was an effect of sex (*F*(1,106) = 7.826, ** *p* = 0.006) with higher freezing levels in males than females. (**B**). Activity levels during the baseline period. There was a genotype x age x sex interaction (*F*(1,106) = 4.620, *p* = 0.034) and in A53T mice, there was an age x sex interaction (*F*(1,65) = 4.843, *p* = 0.032). (**C**). Freezing during the inter-stimulus interval (ISI). There were no significant effects on percent freezing during the ISI. (**D**). Activity levels during the ISI. There was an effect of age (*F*(1,106) = 4.543, * *p* = 0.035), with higher activity levels in older than young mice. In older mice, there was an effect of genotype (*F*(1,67) = 6.163, * *p* = 0.016), with higher activity levels in A54T-L444P than A53T mice. (**E**). Response (motion) to the shocks. There was an effect of age (*F*(1,99) = 8.653, ** *p* = 0.004), with a stronger response in older than younger mice. In young male mice, there was an effect of genotype (*F*(1,22) = 12.229, *p* = 0.002), with lower activity levels in A53T-L444P than A53T male mice.

During the baseline period, there was an effect of sex on the percent freezing (*F*(1,106) = 7.826, *p* = 0.006) and a trend towards a genotype x sex interaction (*F*(1,106) = 3.310, *p* = 0.072) (Figure 5A).Freezing levels were higher in males than in females. When activity levels during the baseline period were analyzed, genotype x age x sex interaction was observed (*F*(1,106) = 4.620, *p* = 0.034) (Figure 5B). In males, there was a trend towards genotype x age interaction (*F*(1,56) = 3.184, *p* = 0.080). In females, there were no significant effects. In A53T mice, age x sex interaction was realized (*F*(1,65) = 4.843, *p* = 0.032). In young A53T mice, there was a trend towards an effect of sex (*F*(1,27) = 3.860, *p* = 0.060). In older A53T mice, this was not seen. In young and older A53T-L444P mice, there were no significant effects.

For freezing during the inter-stimulus intervals (ISIs), a measure of learning or activity levels during the inter-stimulus interval, there were no significant effects (Figure 5C). When activity levels during the ISIs were analyzed, there was a trend towards the effect of age (*F*(1,106) = 3.942, *p* = 0.050). When sex was dropped from the model, age was shown to have an effect (*F*(1,106) = 4.543, *p* = 0.035). In younger mice, there were no significant effects. In older mice, genotype was shown to have an effect (*F*(1,67) = 6.163, *p* = 0.016)

When response (motion) to the shocks was analyzed, age was shown to have an effect (*F*(1,99) = 8.653, *p* = 0.004), with a stronger response in older than in younger mice, along with a sex x genotype x age interaction (*F*(1,99) = 4.280, *p* = 0.041) (Figure 5D). In young mice, there were no significant effects. In older mice, shock was shown to have an effect (*F*(1,64) = 5.513, *p* = 0.022). In young male mice, genotype was shown to have an effect (*F*(1,22) = 12.229, *p* = 0.002). In young female mice, there were no significant effects.

### 3.2. Extinction of Contextual Fear Memory

**Figure 6:**
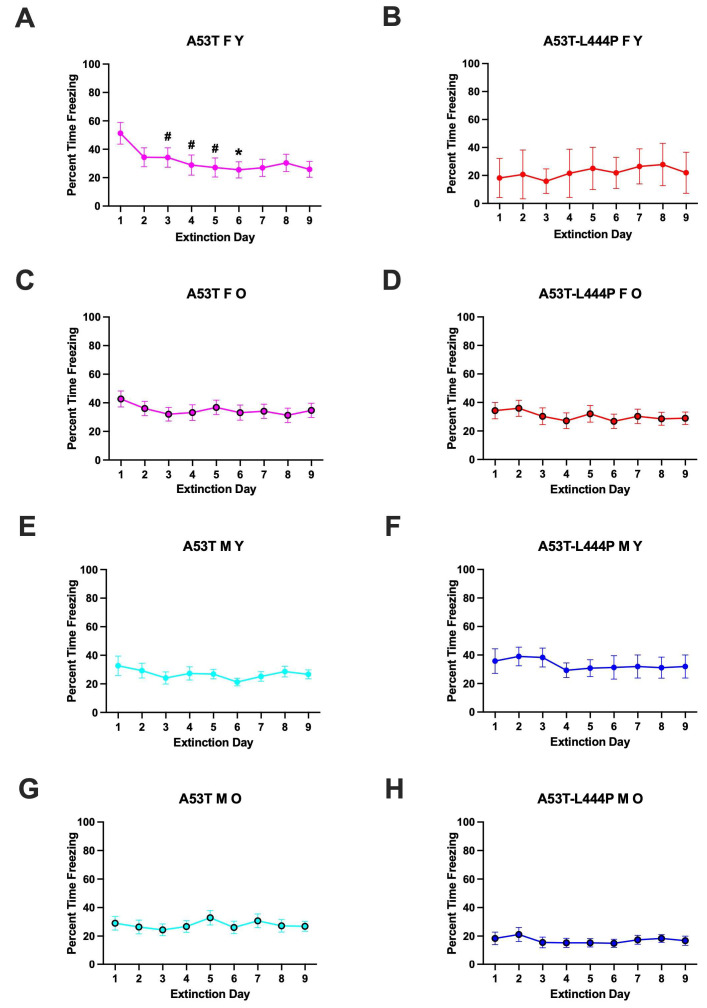
Freezing percentage during the extinction days of young (**A**,**B**,**E**,**F**), older (**C**,**D**,**G**,**H**), female (**A**–**D**), and male (**E**–**H**) mice. (**A**). In young A53T female mice, extinction of contextual fear memory was realized (effect of day: *F*(2.581,28.39) = 3.552, *p* = 0.0320); the freezing levels on day 6 were lower than those on day 1 (* *p* = 0.0270, Dunnett’s), and there was a trend towards lower freezing levels on day 3 (^#^ *p* = 0.0659, Dunnett’s), day 4 (^#^ *p* = 0.0969, Dunnett’s) and day 5 (^#^ *p* = 0.0808, Dunnett’s) compared to those on day 1 (Figure 6A). No extinction of contextual fear memory was seen in any other group.

When percent freezing during the extinction days was analyzed, there was an effect of day (*F*(3.171,313.970) = 3.289, *p* = 0.019 (Greenhouse-Geisser)), a trend towards a sex x age interaction (*F*(1,99) = 3.026, *p* = 0.085). In young A53T female mice, there was extinction of contextual fear memory (effect of day: *F*(2.581,28.39) = 3.552, *p* = 0.0320); the freezing levels on day 6 were lower than those on day 1 (*p* = 0.0270, Dunnett’s) and there was a trend towards lower freezing levels on day 3 (*p* = 0.0659, Dunnett’s), day 4 (*p* = 0.0969, Dunnett’s) and day 5 (*p* = 0.0808, Dunnett’s) than those on day 1 (Figure 6A).No extinction of contextual fear memory was seen in any other group (Figure 6B–H).

When activity levels during the extinction days were analyzed, day x age x sex interaction was observed (*F*(3.254,322.195) = 2.781, *p* = 0.037 (Greenhouse–Geisser)), and there was a trend towards day x genotype interaction (*F*(3.254,322.195) = 2.2193, *p* = 0.083 (Greenhouse–Geisser)). However, there was no effect of day or difference in activity levels on days 2–9 compared to those on day 1 in any group (Figure S2).

When activity levels during the extinction days were analyzed as a percent of baseline activity levels during the first day of extinction, there was an effect of day (*F*(2.252,220.719) = 5.369, *p* < 0.001 (Greenhouse-Geisser)), genotype (*F*(1,98) = 4.088, *p* = 0.046), and a trend towards a day x genotype interaction (*F*(2.252,220.719) = 2.276, *p* = 0.098 (Greenhouse-Geisser)). In A53T mice, there was an effect of day (*F*(2.072,128.438) = 8.182, *p* < 0.001 (Greenhouse-Geisser)), age (*F*(1,62) = 4.655, *p* = 0.035), a day x age interaction (*F*(2.072,128.438) = 3.273, *p* = 0.039 (Greenhouse-Geisser)), a day x sex interaction (*F*(2.072,128.438) = 3.168, *p* = 0.044 (Greenhouse-Geisser)). In A53T-L444P mice, there were no significant effects.

**Figure 7:**
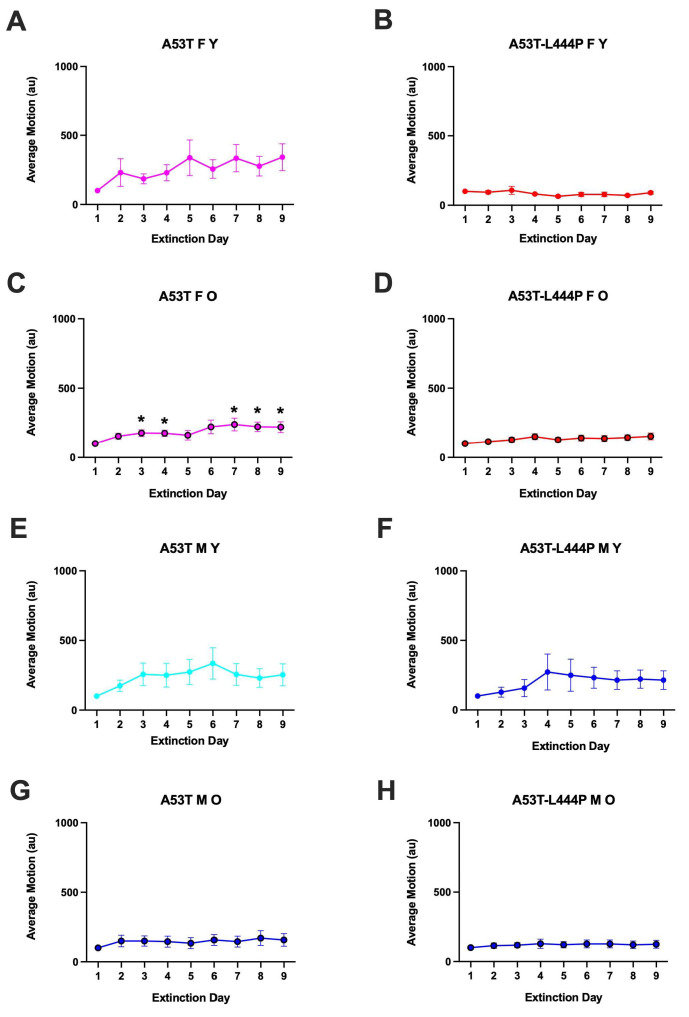
Activity levels as a percent of baseline activity levels on day 1 of extinction of young (**A**,**B**,**E**,**F**), older (**C**,**D**,**G**,**H**), female (**A**–**D**), and male (**E**–**H**) mice. (**C**). In older A53T female mice, there was an effect of day: *F*(2.303,41.46) = 5.421, *p* = 0.0059); the activity levels on days 3 (* *p* = 0.0284, Dunnett’s), 4 (* *p* = 0.0207, Dunnett’s), 7 (* *p* = 0.0415, Dunnett’s), 8 (* *p* = 0.0124, Dunnett’s), and 9 (* *p* = 0.0381, Dunnett’s) were higher than those on day 1 (* *p* = 0.0270, Dunnett’s). (**D**). In older A53T-L444P female mice, there was a trend towards an effect of day *F*(3.328,49.93) = 2.487, *p* = 0.0654) but activity levels on days 2–9 were not different from those on day 1. (**E**). In A53T young male mice, there was a trend towards an effect of day *F*(1.664,24.96) = 5.421, *p* = 0.0681) but activity levels on days 2–9 were not different from those on day 1. In no other group was there an effect of day and activity levels on days 2–9 were not different from those on day 1.

In older A53T female mice, there was an effect of day: *F*(2.303,41.46) = 5.421, *p* = 0.0059); the activity levels on days 3 (*p* = 0.0284, Dunnett’s), 4 (*p* = 0.0207, Dunnett’s), 7 (*p* = 0.0415, Dunnett’s), 8 (*p* = 0.0124, Dunnett’s), and 9 (*p* = 0.0381, Dunnett’s) were higher than those on day 1 (* *p* = 0.0270, Dunnett’s) (Figure 7C). In older A53T-L444P female mice, there was a trend towards an effect of day *F*(3.328,49.93) = 2.487, *p* = 0.0654) but activity levels on days 2–9 were not different from those on day 1 (Figure 7D). In young male A53T mice, there was a trend towards an effect of extinction day *F*(1.664,24.96) = 5.421, *p* = 0.0681), but the activity levels on days 2–9 were not different from those on day 1 (Figure 7E). In no other group was there an effect of day, and activity levels on days 2–9 were not different from those on day 1.

### 3.3. Passive Avoidance Learning

When latency during passive avoidance training was analyzed, there was only a trend towards age as an effect (*F*(1,106) = 3.254, *p* = 0.074) (Figure S2).

### 3.4. Extinction of Passive Avoidance Memory

For latency to enter the dark compartment during the extinction days, there was an effect of day (*F*(4.940,489.050) = 5.310, *p* < 0.001 (Greenhouse-Geisser)), sex (*F*(1,99) = 6.692, *p* = 0.011), a day x age interaction (*F*(4.940,489.050) = 2.764, *p* = 0.018 (Greenhouse-Geisser)), and a sex x genotype interaction (*F*(1,99) = 8.239, *p* = 0.005) (Figure 7). In males, there was an effect of day (*F*(4.854,281.560) = 2.608, *p* = 0.027 (Greenhouse-Geisser)) and a trend towards an effect of genotype (*F*(1,58) = 3.617, *p* = 0.062). In females, there was an effect of day (*F*(4.787,196.255) = 2.942, *p* = 0.015 (Greenhouse-Geisser)) and genotype (*F*(1,41) = 4.734, *p* = 0.035). In A53T mice, there was an effect of day *F*(4.430,287.961) = 3.800, *p* = 0.015 (Greenhouse-Geisser)) and a trend towards an age x sex interaction (*F*(1,65) = 3.190, *p* = 0.079). In A53T-L444P mice, there was an effect of day *F*(4.819,163.853) = 2.650, *p* = 0.026 (Greenhouse-Geisser)), sex (*F*(1,34) = 12.875, *p* = 0.001), and a day x age interaction *F*(4.819,163.853) = 2.533, *p* = 0.033 (Greenhouse-Geisser)).

**Figure 8:**
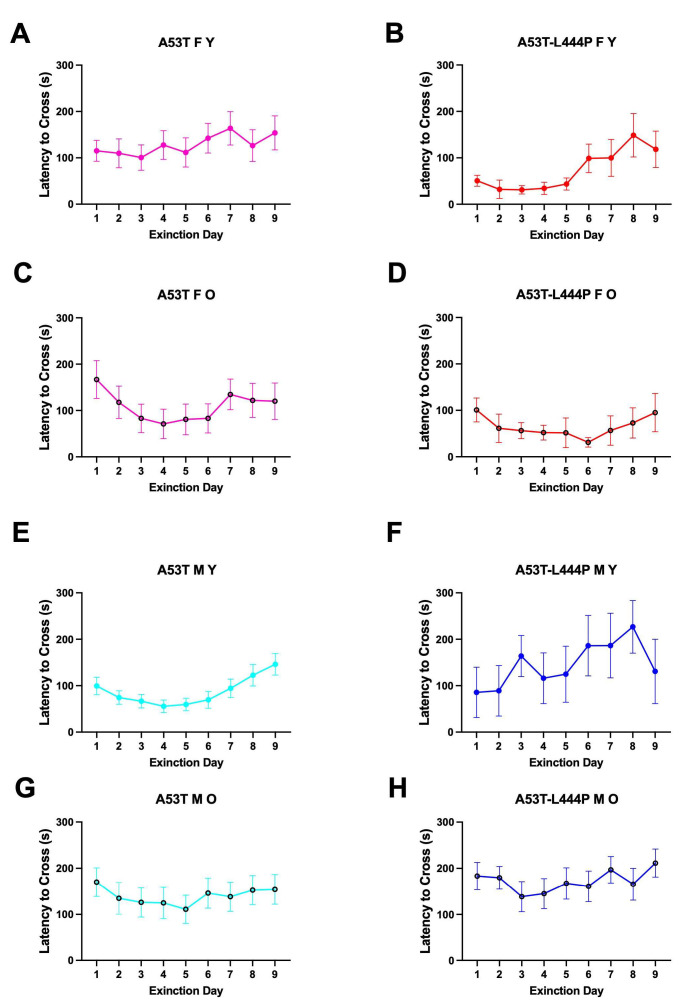
Latency to re-enter the dark compartment during the extinction days of young (**A**,**B**,**E**,**F**), older (**C**,**D**,**G**,**H**), female (**A**–**D**), and male (**E**–**H**) mice. (**B**). there was an effect of day in young A53T-L444P female mice (*F*(3.142,25.13) = 3.255, *p* = 0.0365) but the latency on days 2–9 was not significantly different from that on day 1. **C**). Extinction day was shown to have an effect for young A53T male mice (*F*(4.578,123.6) = 4.679, *p* = 0.0009), but the latency on days 2–9 was not significantly different from that on day 1. Extinction day was not shown to have an effect, and the latency on days 2–9 was not different from that on day 1 in any other group.

When each curve was analyzed separately, there was an effect of day in young A53T-L444P female mice (*F*(3.142,25.13) = 3.255, *p* = 0.0365) (Figure 8B) but the latency on days 2–9 was not significantly different from that on day 1. There was an effect of day in young A53T male mice (*F*(4.578,123.6) = 4.679, *p* = 0.0009) (Figure 8C) but the latency on days 2–9 was not significantly different from that on day 1. There was no effect of day and the latency on days 2–9 was not different from those on day 1 in any other group.

We next analyzed the extinction curves as percent of baseline compared to the latency of the mouse on the first day of extinction. There was an effect of day (*F*(2.339,321.567) = 6.801, *p* < 0.001 (Greenhouse-Geisser)), a trend towards an effect of age (*F*(1,99) = 3.458, *p* = 0.066), and a trend towards a sex x genotype interaction (*F*(1,99) = 3.394, *p* = 0.068) (Figure 8). In males, there was an effect of day (*F*(3.205,185.891) = 4.978, *p* = 0.002), age (*F*(1,58) = 5.343, *p* = 0.024), and genotype (*F*(1,58) = 4.774, *p* = 0.033). In male A53T mice, there was an effect of day (*F*(2.293,116.904) = 4.819, *p* = 0.004 (Greenhouse-Geisser)) (Figure 8C,G)). In male A53T-L444P mice, there was also an effect of day (*F*(3.321,59.784) = 2.800, *p* = 0.043 (Greenhouse-Geisser)) and a trend towards an effect of age (*F*(1,18) = 3.637, *p* = 0.073) (Figure 8D,H). In females, there was only a trend towards an effect of day (*F*(1.764,72.305) = 2.685, *p* = 0.082 (Greenhouse-Geisser)). In A53T females, there was an effect of day *F*(2.002,130.107) = 5.395, *p* = 0.006 (Greenhouse-Geisser)).

In A53T mice, there was an effect of day (*F*(2.002,130.107) = 5.395, *p* = 0.006 (Greenhouse-Geisser)). In A53T-L444P mice, there was an effect of age (*F*(1,34) = 4.833, *p* = 0.035), day (*F*(3.128,106.344) = 3.383, *p* = 0.019 (Greenhouse-Geisser)), a trend towards an effect of sex (*F*(1,34) = 3.682, *p* = 0.063), and a trend towards a day x age interaction (*F*(1,34) = 3.682, *p* = 0.063), day (*F*(3.128,106.344) = 2.355, *p* = 0.075 (Greenhouse-Geisser)).

**Figure 9:**
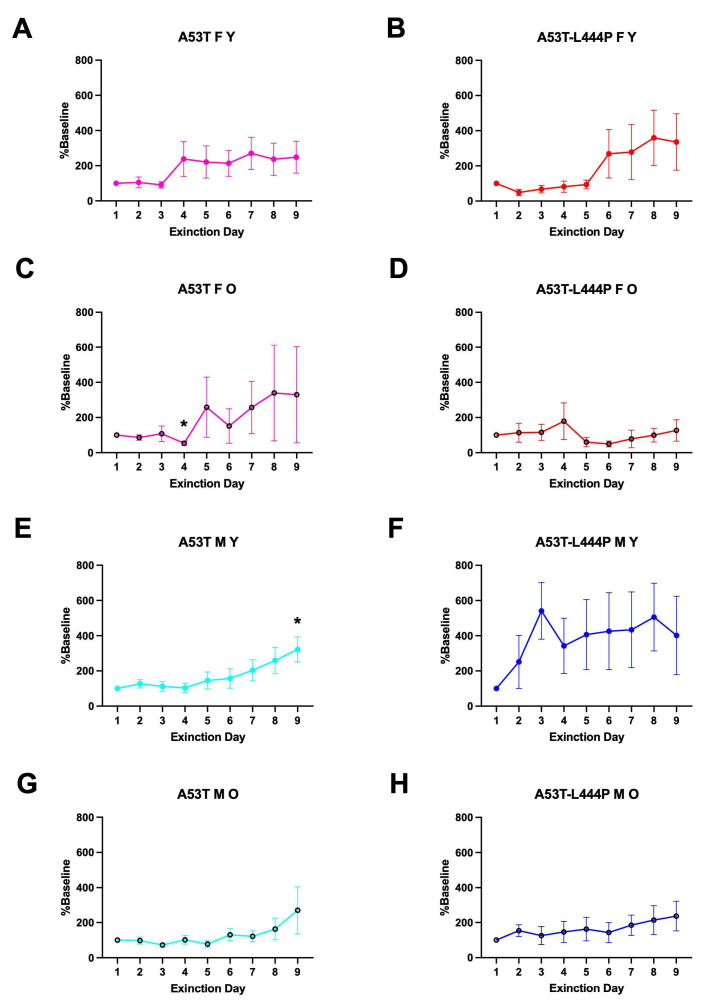
Latency to re-enter the dark compartment expressed as percent of baseline latency on day 1 of extinction of young (**A**,**B**,**E**,**F**), older (**C**,**D**,**G**,**H**), female (**A**–**D**), and male (**E**–**H**) mice. (**C**). In older A53T female mice, there was no effect of day but the percent latency was lower on day 4 than day 1 (* *p* = 0.0149, Dunnett’s). (**E**). In young A53T male mice, there was an effect of day (*F*(2.719,73.41) = 4.643, *p* = 0.0064), and the percent latency was higher on day 9 than day 1 (* *p* = 0.0267, Dunnett’s) (Figure 8E). There was no effect of day and the percent latency on days 2–9 was not different than those on day 1 in any other group.

In older A53T female mice, there was no effect of day but the percent latency was lower on day 4 than day 1 (*p* = 0.0149, Dunnett’s) (Figure 9C). In young A53T male mice, there was an effect of day (*F*(2.719,73.41) = 4.643, *p* = 0.0064), and the percent latency was higher on day 9 than day 1 (*p* = 0.0267, Dunnett’s) (Figure 9E).

As none of the groups showed extinction of passive avoidance memory, and due to the complexity of the model and the genetic background, we assessed whether C57BL/6J wild-type female and male mice show extinction of passive avoidance memory. There was no sex difference in latency to enter the dark compartment on the training day (*F* = 2.075, *p* = 0.2021 (2-tailed *t* test) (Figure S3A). There was no extinction of passive avoidance memory in C57BL/6J wild-type female (Figure S3B) and male (Figure S3C) (*F*(4,112) = 0.137, *p* = 0.969) mice, and no sex difference either (*F*(1,28) = 2.203, *p* = 0.149).

### 3.5. Performance in the Open Field

**Figure 10:**
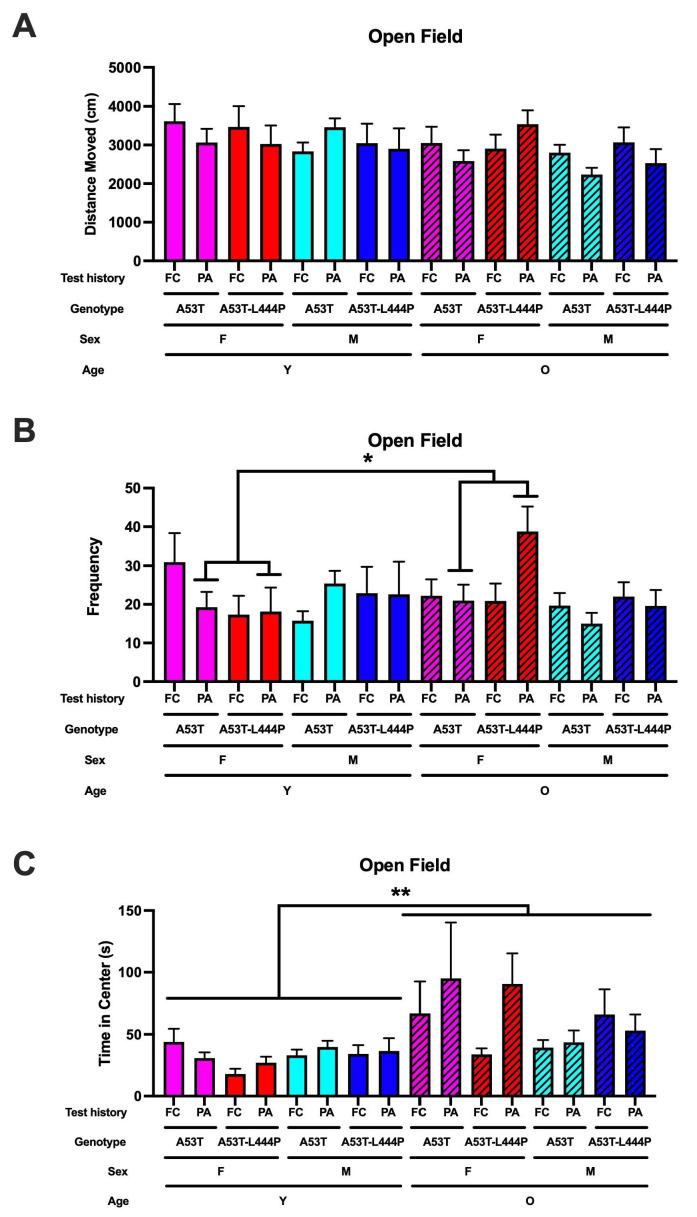
(**A**). Activity levels in the open field were analyzed. (**B**). Frequency to enter the more anxiety-provoking center of the open field. For activity levels in the open field, there was a trend towards an effect of age (*F*(1,209) = 2.765, *p* = 0.098) and a trend towards a test history x sex x age interaction (*F*(1,209) = 2.848, *p* = 0.093). (**B**). Frequency to enter the more anxiety-provoking center of the open field. There was a test history x sex x age interaction (*F*(1,209) = 4.362, *p* = 0.038) and a trend towards a test history x sex genotype interaction (*F*(1,209) = 3.488, *p* = 0.063). In mice that had received extinction of passive avoidance memory, there was a sex x age interaction (*F*(1,102) = 6.461, *p* = 0.013) and a trend towards a genotype x age interaction (*F*(1,102) = 3.525, *p* = 0.064). In female mice that had received extinction of passive avoidance memory, there was an effect of age (*F*(1,40) = 4.803, * *p* = 0.035). (**C**). Time spent in the center of the open field. There was an effect of age (*F*(1,94) = 8.214, ** *p* = 0.005), with older mice spending more time in the center than younger mice.

For activity levels in the open field, there was a trend towards an effect of age (*F*(1,209) = 2.765, *p* = 0.098) and a trend towards a test history x sex x age interaction (*F*(1,209) = 2.848, *p* = 0.093) (Figure 10A).

When frequency to enter the more anxiety-provoking center of the open field was analyzed, there was a test history x sex x age interaction (*F*(1,209) = 4.362, *p* = 0.038) and a trend towards a test history x sex genotype interaction (*F*(1,209) = 3.488, *p* = 0.063) (Figure 10B). In mice that had received extinction of fear conditioning, there were no significant effects. In mice that had received extinction of passive avoidance memory, there was a sex x age interaction (*F*(1,102) = 6.461, *p* = 0.013) and a trend towards a genotype x age interaction (*F*(1,102) = 3.525, *p* = 0.064). In male mice that had received extinction of passive avoidance memory, there were no significant effects. In female mice that had received extinction of passive avoidance memory, there was an effect of age (*F*(1,40) = 4.803, *p* = 0.035) and a trend towards a genotype x age interaction (*F*(1,40) = 3.476, *p* = 0.070).

For time spent in the more anxiety-provoking center of the open field, there was an effect of age (*F*(1,94) = 8.214, *p* = 0.005) (Figure 10C).

### 3.6. Performance in the Forced Swim Test

**Figure 11:**
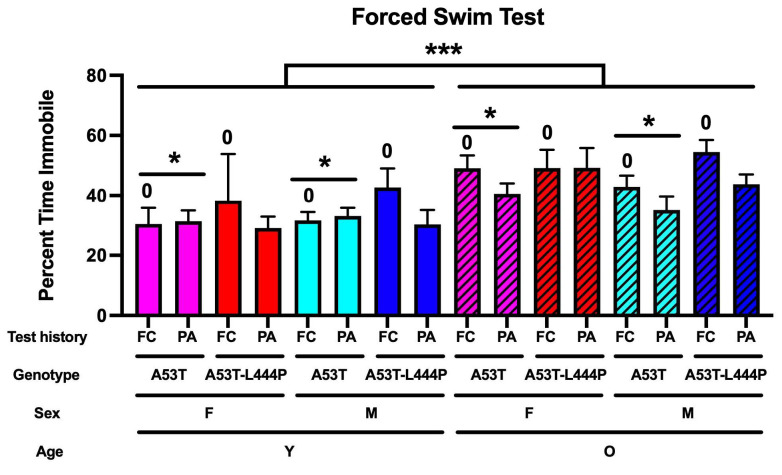
Depressive-like behavior in the forced swim test. For depressive-like behavior in the forced swim test, test history was shown to have an effect (*F*(1,213) = 5.040, ^0^ *p* = 0.026), with more depressive-like behavior in mice trained for acquisition and extinction of contextual fear memory (FC) than in mice trained for passive avoidance memory extinction (PA). Age was shown to have an effect (*F*(1,213) = 22.362, *** *p* < 0.001), with more depressive-like behavior in older than in younger mice. Genotype was also shown to have an effect (*F*(1,213) = 4.344, * *p* = 0.038), with more depressive-like behavior in A53T-L444P than in A53T mice.

For depressive-like behavior in the forced swim test, test history was shown to have an effect (*F*(1,213) = 5.040, *p* = 0.026), with more depressive-like behavior in mice trained for acquisition and extinction of contextual fear memory than in mice trained for passive avoidance learning and memory extinction. Age was also shown to have an effect (*F*(1,213) = 22.362, *p* < 0.001), with more depressive-like behavior in older than in younger mice; genotype was also shown to have an effect (*F*(1,213) = 4.344, *p* = 0.038) (Figure 11).

### 3.7. Phosphorylated Tau Data

**Figure 12:**
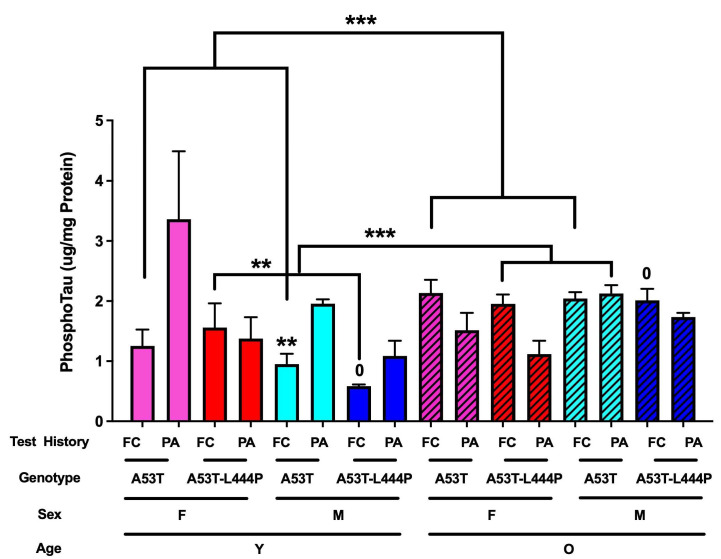
Phosphorylated tau levels in the cortex. In A53T mice that had received extinction of fear conditioning memory, there was an effect of age (*F*(1,31) = 7.218, *** *p* < 0.001). In A53T-L444P mice that had received extinction of fear conditioning memory, there was an effect of sex (*F*(1,27) = 4.821, *p* = 0.038) and an effect of age (*F*(1,27) = 19.123, *** *p* < 0.001). In young A53T-L444P mice that had received extinction of fear conditioning memory, there was an effect of sex (*F*(1,8) = 13.415, ** *p* = 0.008). In A53T-L444P male mice that had received extinction of fear conditioning memory, there was an effect of age (*F*(1,14) = 36.097, ^0^ *p* < 0.001).

When the cortical phosphorylated tau (pS 199) levels were analyzed, there was an effect of genotype (*F*(1,118) = 6.741, *p* = 0.011), a sex x age interaction (*F*(1,118) = 7.647, *p* = 0.007), and an age x test history interaction (*F*(1,118) = 11.393, *p* = 0.001). In A53T mice, there was an age x test history interaction (*F*(1,62) = 8.090, *p* = 0.006), a trend towards an effect of test history (*F*(1,118) = 4.017, *p* = 0.050), and a trend towards a sex x age interaction (*F*(1,118) = 3.003, *p* = 0.089) (Figure 12). In A53T mice that had received extinction of fear conditioning memory, there was an effect of age (*F*(1,31) = 7.218, *p* < 0.001). In A53T mice that had received extinction of passive avoidance memory, there were no significant effects. In A53T-L444P mice that had received extinction of fear conditioning memory, there was an effect of sex (*F*(1,27) = 4.821, *p* = 0.038), an effect of age (*F*(1,27) = 19.123, *p* < 0.001) and a sex x age interaction (*F*(1,27) = 6.176, *p* = 0.020). In A53T-L444P mice that had received extinction of passive avoidance memory, there was a trend towards a sex x age interaction (*F*(1,27) = 4.005, *p* = 0.057). In young and older A53T mice that had received extinction of fear conditioning memory, there were no significant effects. In young A53T-L444P mice that had received extinction of fear conditioning memory, there was an effect of sex (*F*(1,8) = 13.415, *p* = 0.008). In older A53T-L444P mice that had received extinction of fear conditioning memory, there were no significant effects. In A53T-L444P male mice that had received extinction of fear conditioning memory, there was an effect of age (*F*(1,14) = 36.097, *p* < 0.001). In A53T-L444P female mice that had received extinction of fear conditioning memory, there were no significant effects.

There was a weak positive correlation between phosphorylated tau levels and immobility percentage in the forced swim test (*r* = 0.2089, *p* = 0.0226, Pearson, *n* = 119) (Figure S4A). As there were only three data points with phosphorylated tau levels above 4 mg/mg protein, we repeated this analysis with these three data points removed. This resulted in a higher r value and more significance (*r* = 0.2542, *p* = 0.0059, Pearson, *n* = 116) (Figure S4B).

## 4. Discussion

This study supports hypothesis that both A53T and A53T-L444P affect the risk of developing PTSD phenotypes. Only young female A53T mice showed contextual fear memory extinction, while female A53T and female and male A53T-L444P mice did not. The fact that younger A53T-L444P mice did not show extinction of contextual fear memory is consistent with the hypothesis that A53T-L444P is a more of a risk factor for PTSD phenotypes than A53T alone. As the risk of developing PTSD is 2–3-fold higher in women than in men [17], and the risk of developing PD is 2-fold higher in men than in women [18], the extinction of contextual fear memory in younger A53T female—but not male—mice suggests that in the context of PD, male sex might be a stronger risk factor than A53T for developing PTSD phenotypes. Consistent with this pattern, men have a higher risk of developing asynocleinopathies and with an earlier age at onset than women [40]. In addition, in patients with advanced PD, plasma alpha synuclein levels were lower in males than in females; moreover, in males, plasma alpha synuclein levels were associated with cognitive impairments, hallucinations, and sleep disorders [41]. Interestingly, sex differences are not seen in patients with Gaucher disease [42,43]. The data from our study suggest that while, in general, females have a higher risk of developing PTSD than males [17], PTSD phenotypes might be similar in the presence of L444P, and only seen in the presence of A53T in the absence of L444P.

The lack of extinction in contextual fear memory in the young A53T male mice and young and older A53T-L444P female and male mice is dramatic. In young and middle-aged human apolipoprotein E3 mice, extinction of contextual fear memory is already seen within the first three days of extinction training. Consistent with the human apolipoprotein E3 mice, in C57BL6/J wild-type mice, extinction of contextual fear memory is seen within the first five days of extinction [44]. The human apolipoprotein E3 mice are on a C57BL6/J background, a strain that performs well in contextual fear learning and memory tests [45,46,47,48]. We recognize that the background strain of the A53T and A53T-L444P mice might have contributed to the dramatic PTSD phenotype seen in contextual fear memory extinction.

The lack of extinction of passive avoidance memory in the A53T and A53T-L444P mice is even more dramatic. In no group is extinction in the passive avoidance memory paradigm seen. When the data are normalized to the first day of extinction, in older A53T female mice, a lower latency percentage is seen on day 4 compared to day 1; however, on subsequent days, the values increase. In contrast, in middle-aged, but not young, apolipoprotein E3 mice, extinction of passive avoidance memory is already seen on the second day of extinction. The background strain not being C57BL/6J likely did not contribute to the dramatic phenotype in passive avoidance memory extinction. In female and male C57BL/6J mice tested for extinction of passive avoidance memory, no extinction was seen in female or male mice, and no sex differences were seen either. In contrast, in other studies, C57BL/6J and AKR/J show a faster extinction of passive avoidance memory than DBA/2J, CBA/Lac, BALB/c, and C3H/HeJ mice [49]; in C57BL/6J mice, females show a slower extinction of passive avoidance memory than male mice [50]. Differences in experimental paradigms or environmental conditions might have contributed to these divergent findings.

The open field and forced swim test data support that having been exposed to the contextual fear memory versus the passive avoidance extinction paradigm affects measures of anxiety and depressive-like behavior. For frequency to enter the more anxiety-provoking center of the open field, there was a test history x sex x age interaction. In the forced swim test, there was an effect of test history with more depressive-like behavior in mice trained in the contextual fear memory extinction paradigm than in mice trained in the passive avoidance memory extinction paradigm. In addition to the effects of test history, there was an effect of age with more depressive-like behavior in older than younger mice, and an effect of genotype with more depressive-like behavior in A53T-L444P than in A53T mice. Depression is a common symptom in PD patients [51] and in PD animal models [52,53]; often, there is hesitation to prescribe antidepressants to PD patients because of potential interactions between antidepressants and anti-movement-related symptoms [54].

The cortical phosphorylated tau data support that having been exposed to the contextual fear memory versus the passive avoidance extinction paradigm affects PD-related neuropathology as well. When cortical phosphorylated tau (pS 199) levels were analyzed, there were effects of genotype, a sex x age interaction, and an age x test history interaction. In young mice, besides A53T-L444P female mice, higher phosphorylated tau levels were seen in mice that had received the extinction of passive avoidance memory paradigm than those that had received the extinction of contextual fear memory paradigm. In contrast, in older mice, besides A53T male mice, lower phosphorylated tau levels were seen in mice that had received the extinction of passive avoidance memory paradigm than those that had received the extinction of contextual fear memory paradigm.

L444P heterozygous mice show hippocampus-dependent cognitive injury independent of alpha synuclein accumulation, motor deficits, and dopaminergic dysfunction; this might involve a reduction in the density of CA3-CA1 synapses and impaired basal synaptic transmission and plasticity [55]. When expressed together with wild-type human alpha synuclein, hippocampal accumulation of alpha synuclein is increased and associated with more severe hippocampus-dependent cognitive injury, hippocampal plasticity, and motor impairments [55]. The L444P mutation might affect the aggregation of alpha synuclein via mitochondrial dysfunction, disturbances in lipid homeostasis, impaired trafficking from the endoplasmatic reticulum to the lysosome, and neuroinflammation [56]. The impairment in extinction of hippocampus-dependent contextual fear memory in A53T-L444P but not in A53T female mice, in the absence of genotype differences in phosphorylated tau levels, is consistent with the notion that L444P can have detrimental effects on hippocampal function independent of the accumulation of alpha synuclein [55].

These data highlight the importance of considering to include avoidance in fear memory extinction. Future efforts are warranted to determine the pathways which might be engaged during and following extinction of passive avoidance memory versus extinction of contextual fear memory that might be targeted to reduce measure of anxiety and depressive-like behavior and tau pathology in PD and other neurological conditions.

## Data Availability

The data supporting the findings of this study are available within the article and its Supplementary Material.

## Supplementary Materials

The following supporting information can be downloaded at: https://www.mdpi.com/article/10.3390/genes16091004/s1, Figures S1–S4. Figure S1. Activity levels of young (A, B, E, F), older (C, D, G, H), female (A, B, C, D), and male (E, F, G, H) mice during the days of extinction. When activity levels during the extinction days were analyzed, there was a day x age x sex interaction (*F*(3.254,322.195) = 2.781, *p* = 0.037 (Greenhouse-Geisser)) and there was a trend towards a day x genotype interaction (*F*(3.254,322.195) = 2.2193, *p* = 0.083 (Greenhouse-Geisser)). However, there was no effect of day or difference in activity levels on days 2–9 compared to those on day 1 in any group. Figure S2. Latency during passive avoidance training. Figure S3. A. Latency of C57BL/6J (WT) female (F) and male (M) mice during passive avoidance training. B. Latency of female WT mice to re-enter the dark compartment during extinction days. C. Latency of male WT mice to re-enter the dark compartment during extinction days. Figure S4. A. A weak positive correlation between phosphorylated tau levels and percent immobility in the forced swim test (*r* = 0.2089, *p* = 0.0226, Pearson, *n* = 119). B. We repeated this analysis with the three data points with phosphorylated tau levels higher than 4 mg/mg protein removed. This resulted in a higher r value and more significance (*r* = 0.2542, *p* = 0.0059, Pearson, *n* = 116).
