# Supplementary material for: Extinction of Contextual Fear Memory and Passive Avoidance Memory and Subsequent Anxiety-like and Depressive-like Behavior of A53T and A53T-L444P Mice
Source: Genes (Basel). 2025 Aug 26;16(9):1004. doi: 10.3390/genes16091004 (PMC12470044; doi:10.3390/genes16091004)
Supplement: Supplementary file 1 [file genes-16-01004-s001.zip › genes-3806392-supplementary.pdf]

Supplement Paper

Extinction of contextual fear memory and passive avoidance memory and subsequent anxiety-like and depressive-like behavior of A53T and A53T-L444P Mice

Emily Bunnell[1*], Elizabeth Saltonstall[1*], Alexandra Pederson[1], Charlie Baxter[1], Elia Ramicciotti[1], Naomi Robinson[1], Phoebe Sandholm[1], Abigail O'Niel[1], Jacob Raber[1,2,3#]

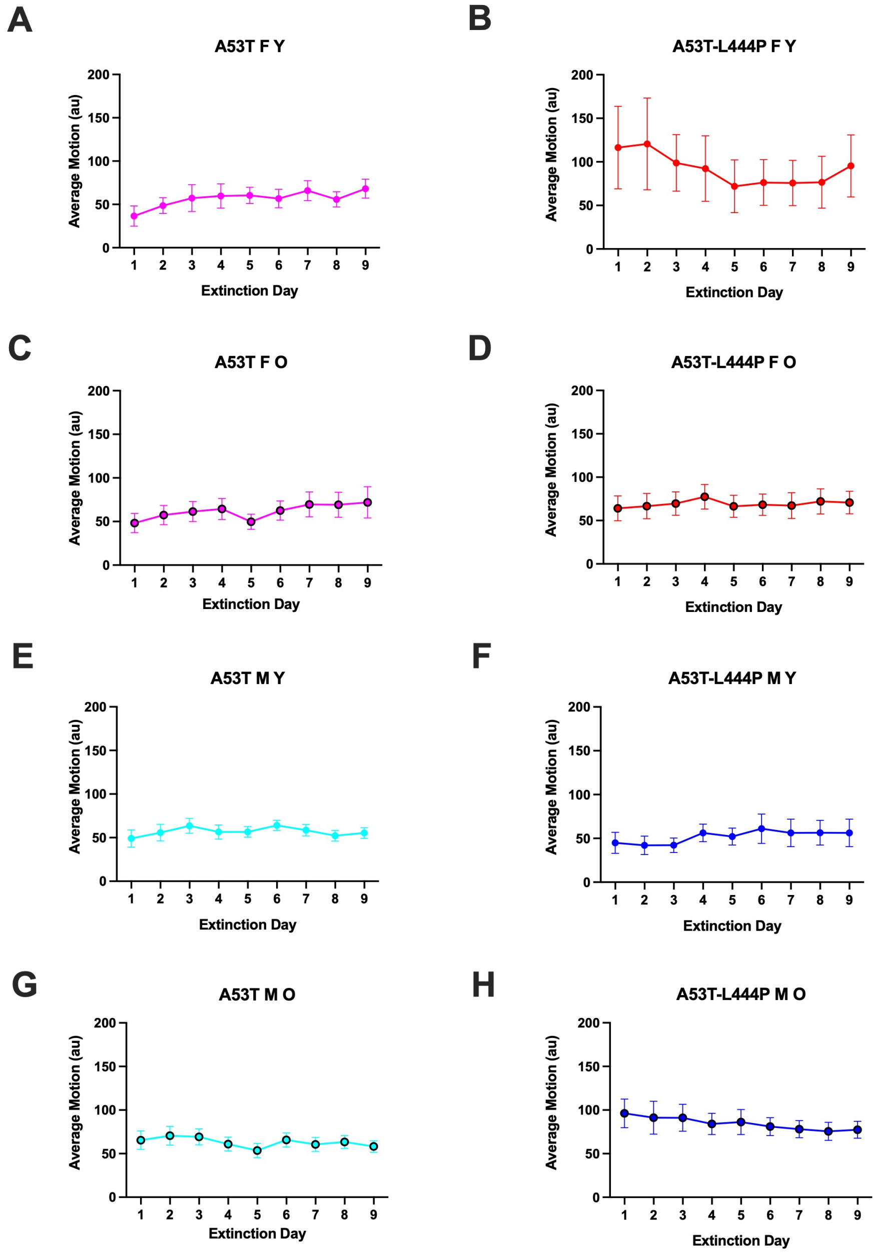

Fig. S1. Activity levels of young (A, B, E, F), older (C, D, G, H), female (A, B, C, D), and male (E, F, G, H) mice during the days of extinction. When activity levels during the extinction days were analyzed, there was a day x age x sex interaction ($F(3.254,322.195) = 2.781$, $p = 0.037$ (Greenhouse-Geisser)) and there was a trend towards a day x genotype

interaction ($F(3.254,322.195) = 2.2193$, $p = 0.083$ (Greenhouse-Geisser)). However, there was no effect of day or difference in activity levels on days 2-9 compared to those on day 1 in any group.

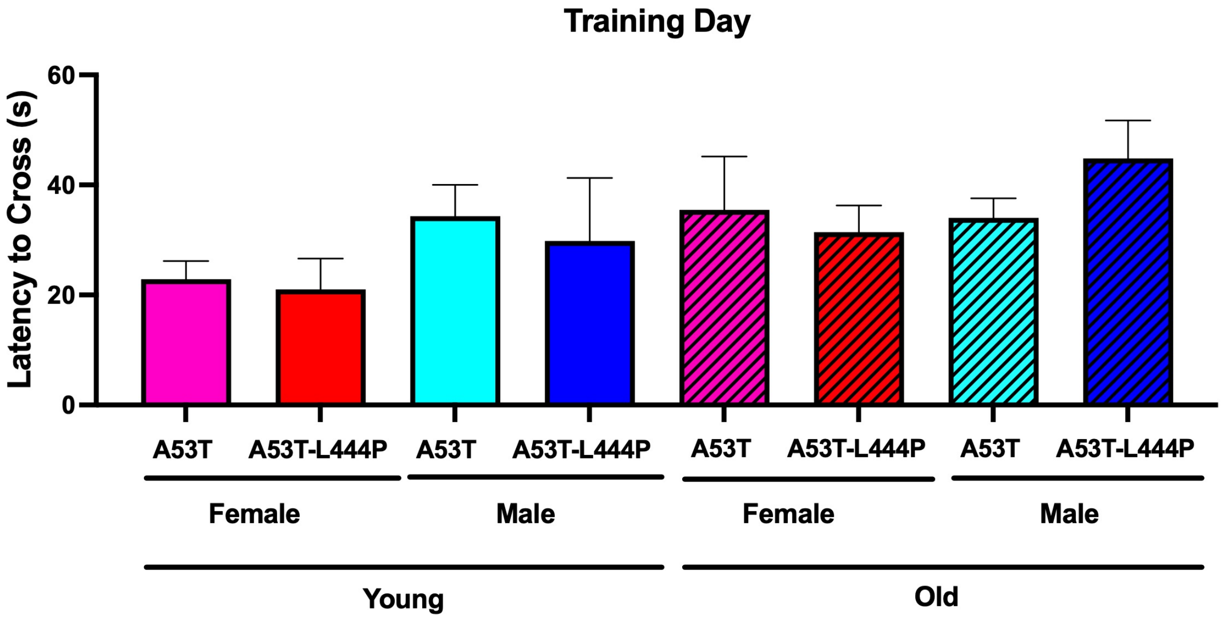

Fig. S2. Latency during passive avoidance training.

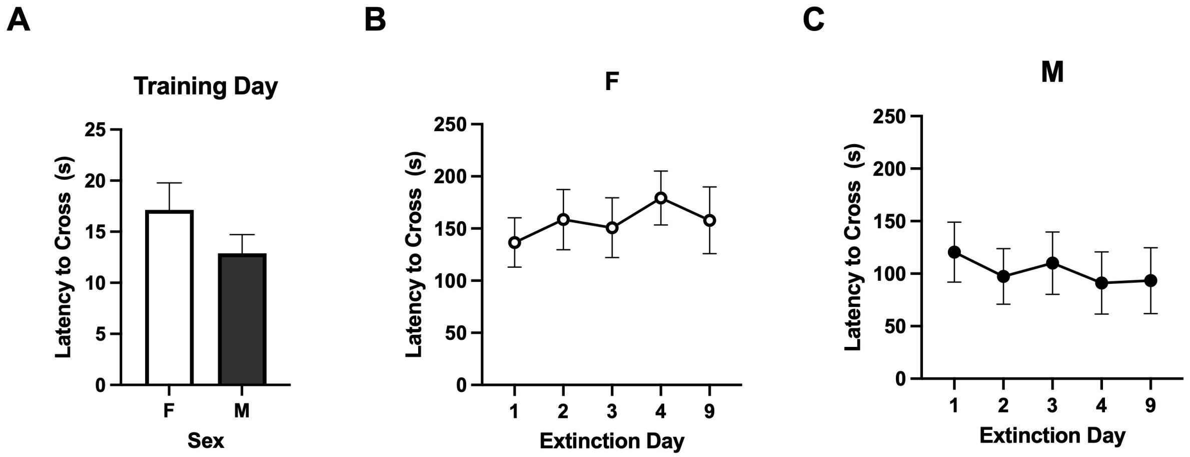

Fig. S3. A. Latency of C57BL/6J (WT) female (F) and male (M) mice during passive avoidance training. **B.** Latency of female WT mice to re-enter the dark compartment during extinction days. **C.** Latency of male WT mice to re-enter the dark compartment during extinction days.

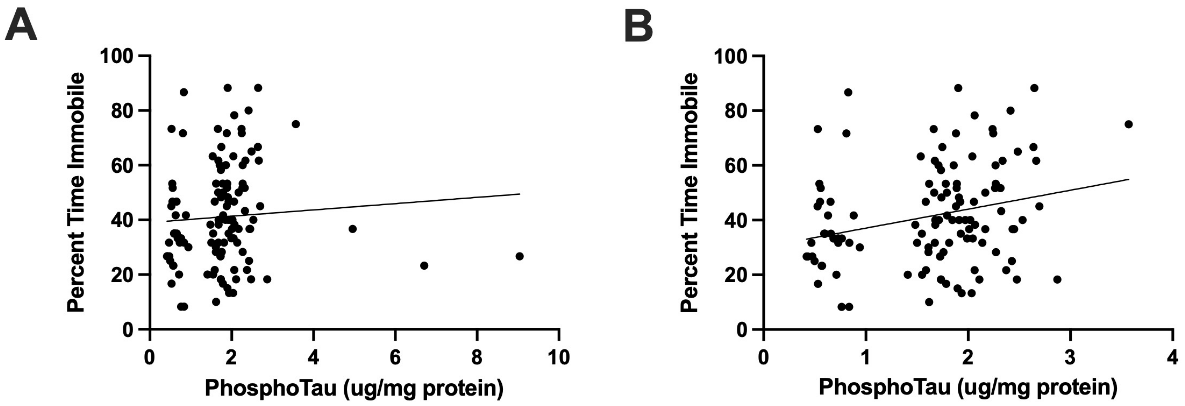

Fig. S4. A. A weak positive correlation between phosphorylated tau levels and percent immobility in the forced swim test (r = 0.2089, p = 0.0226, Pearson, n = 119). **B.** We repeated this analysis with the three data points with phosphorylated tau levels higher than 4 μg/mg protein removed. This resulted in a higher r value and more significance (r = 0.2542, p = 0.0059, Pearson, n = 116).
